# Discovery of broadly‐neutralizing antibodies against brown recluse spider and Gadim scorpion sphingomyelinases using consensus toxins as antigens

**DOI:** 10.1002/pro.4901

**Published:** 2024-02-15

**Authors:** Esperanza Rivera‐de‐Torre, Stefanos Lampadariou, Mark Møiniche, Markus F. Bohn, Seyed Mahdi Kazemi, Andreas H. Laustsen

**Affiliations:** ^1^ Department of Biotechnology and Biomedicine Technical University of Denmark Kongens Lyngby Denmark; ^2^ Zagros Herpetological Institute Qom Iran

**Keywords:** antivenom, broadly‐neutralizing antibodies, consensus antigen, consensus toxin, *Hemiscorpius*, *Loxosceles*, phage display, recluse spider, sphingomyelinase, toxins

## Abstract

Broadly‐neutralizing monoclonal antibodies are becoming increasingly important tools for treating infectious diseases and animal envenomings. However, designing and developing broadly‐neutralizing antibodies can be cumbersome using traditional low‐throughput iterative protein engineering methods. Here, we present a new high‐throughput approach for the standardized discovery of broadly‐neutralizing monoclonal antibodies relying on phage display technology and consensus antigens representing average sequences of related proteins. We showcase the utility of this approach by applying it to toxic sphingomyelinases from the venoms of species from very distant orders of the animal kingdom, the recluse spider and Gadim scorpion. First, we designed a consensus sphingomyelinase and performed three rounds of phage display selection, followed by DELFIA‐based screening and ranking, and benchmarked this to a similar campaign involving cross‐panning against recombinant versions of the native toxins. Second, we identified two scFvs that not only bind the consensus toxins, but which can also neutralize sphingomyelinase activity of native whole venom in vitro. Finally, we conclude that the phage display campaign involving the use of the consensus toxin was more successful in yielding cross‐neutralizing scFvs than the phage display campaign involving cross‐panning.

## 1 INTRODUCTION

Monoclonal antibodies (mAbs) are the largest group of biotherapeutic agents used to treat diseases, with more than 100 of such molecules approved for clinical applications (Kaplon et al., 2022). However, for some applications, such as controlling infectious diseases or neutralizing toxins, the high specificity of mAbs comes with the disadvantage that the binding properties of mAbs may not always be robust against antigenic variation (Salazar et al., 2017). This limitation has recently been a particular concern for mAbs targeting escape mutants of SARS‐CoV2 (Wilhelm et al., 2022), but antigenic variation has for more than a century also complicated the development of envenoming therapies, where antibodies need to neutralize large numbers of similar and dissimilar toxins (Casewell et al., 2020). Broadly‐neutralizing human mAbs could serve as useful tools in this regard due to their compatibility with the human immune system and their versatility across indications. Yet, better methods are needed to discover such antibodies that can bind more than a single protein isoform or epitope, if we are to combat rapidly evolving or multi‐component diseases.

Recently, it was demonstrated that broadly‐neutralizing mAbs (bnAbs) targeting animal toxins can be developed using cross‐panning strategies in phage display selection campaigns (Ahmadi et al., 2020; Ledsgaard et al., 2023; Sørensen et al., 2023), by semi‐rational design and directed evolution (Rodríguez‐Rodríguez et al., 2016), and by high‐throughput screening of B‐cells from immunized individuals (Glanville et al., 2022). Here, we demonstrate the utility of a fourth strategy that uses consensus toxins, which are artificial toxins based on the average sequence of related toxins (Rivera‐de‐Torre et al., 2022). These have previously been used as immunogens to generate broadly‐neutralizing polyclonal antibody responses in animals (de la Rosa et al., 2018; de la Rosa et al., 2019). However, the objective of this study was to explore the utility of consensus antigens in an in vitro display technology‐based antibody discovery campaign to discover bnAbs (Laustsen et al., 2021; Ledsgaard et al., 2022). This was achieved by designing a consensus toxin and using phage display selection to discover bnAbs with cross‐reactivity against toxins from two distantly related species deriving from distant orders of the animal Kingdom, namely *Loxosceles* sp. (recluse spiders) and *Hemiscorpius* sp. (Gadim scorpions) found in North Africa and the Middle East, respectively (Jenkins et al., 2021), and comparing this discovery campaign to one utilizing a previously established cross‐panning strategy (Ahmadi et al., 2020).

Both recluse spiders and Gadim scorpions are of medical importance due to the high incidence of spider bites and scorpion stings in the regions they are endemic in. In Iran alone, between 40,000 and 50,000 cases of scorpion stings occur annually, resulting in approximately 20 deaths, with *H. lepturus* responsible for 10%–25% of all sting cases and nearly 70% of the fatalities (Dehghani and Fathi, 2012). Unlike other scorpion stings, *Hemiscorpius* sp. stings are not immediately painful, but result in delayed necrotoxic and hemotoxic symptoms that resemble those caused by *Loxosceles* sp. bites. Due to the delay in the onset of clinical manifestations, victims may delay seeking medical attention, which in turn contributes to the high mortality rate associated with *Hemiscorpius* sp. stings. The primary toxin responsible for the clinical manifestations observed for *Hemiscorpius* sp. stings is a sphingomyelinase (SMase), for which related isoforms also exist in *Loxosceles* sp. venom. Evolutionary analysis of these toxic SMases reveals that they are produced by human pathogenic bacteria (*Corynebacterium pseudotuberculosis* and *Arcanobacterium haemolyticum*), ticks (*Ixodes scapularis* and *Rhipicephalus pulchellus*), as well as spiders (*Loxosceles* sp. and *Sicarius* sp.) and scorpions (*Centuroides* sp. and *Hemiscorpius* sp.) (Pedroso et al., 2015). SMases cause cytotoxicity by transforming sphingomyelin in cellular membranes into ceramide and phosphorylcholine, thereby disrupting the membranes and triggering necrosis through ceramide‐induced apoptosis (Catalán et al., 2011; de Oliveira et al., 2005; Seyedian et al., 2010). Therefore, neutralizing these SMases quickly after an envenoming has occurred is of high therapeutic relevance to achieve an optimal treatment outcome for spider bite and scorpion sting victims.

In this work, we designed a consensus SMase (cSMase) that is an artificial toxin resembling an average sequence and structure of both *Loxosceles* sp. and *Hemiscorpius* sp. SMases. We decided to restrict the design to only incorporate SMases from scorpions and spiders, as only these SMases are relevant for envenoming cases. We further demonstrate the advantage of using our designed cSMase as an antigen in a phage display selection campaign aimed at discovering single‐chain variable fragments (scFvs) that bind and neutralize native SMases from both species. By comparing the utility of a stringent selection strategy using the cSMase as an antigen with a strategy involving cross‐panning using recombinant versions of the native toxins, we find that the strategy involving the consensus toxin was more successful, as it yielded scFvs that bind to natural SMases from *H. lepturus* and *L. rufescens* with higher affinity than those scFvs identified using the cross‐panning strategy. Furthermore, we demonstrate that the scFvs obtained using the cSMase bind to SMases from *H. lepturus* and *L. rufescens* with similar affinity and can effectively neutralize the SMase activity of *H. lepturus* whole venom in vitro. These results thus provide evidence for the potential utility of using consensus antigens and phage display technology as a generalizable strategy for the discovery of broadly‐neutralizing antibodies, as well as the study presents specific molecules that could be subject for further preclinical development.

## 2 RESULTS

**FIGURE 1:**
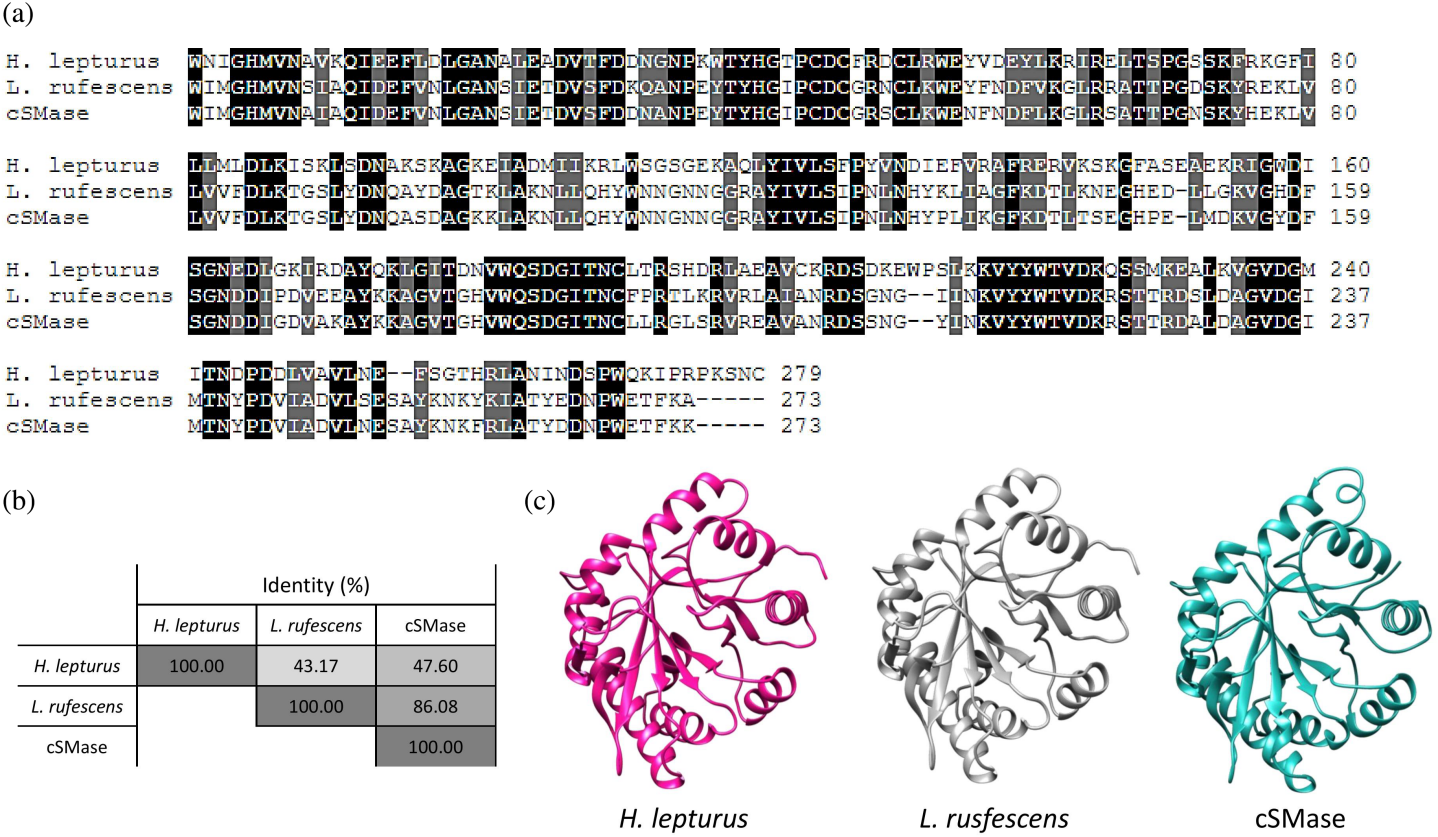
Comparison of SMases from *H. lepturus* (A0A1L4BJ98), *L. rufescens* (C0JB02.1), and the designed consensus SMase (cSMase). (a) Sequence alignment was performed using Clustal Omega default settings, and the resulting alignment was color‐coded to indicate the degree of conservation for each position. A black shade indicates a completely conserved amino acid, while a gray shade indicates conservation of physicochemical properties. White indicates non‐conserved amino acids. (b) The percentage identity matrix shows the level of similarity between the aligned proteins. The values are indicated as percentages and are displayed in a color‐coded matrix. (c) The predicted three‐dimensional structure of cSMase was generated using AlphaFold2. SMase, sphingomyelinase.

**FIGURE 2:**
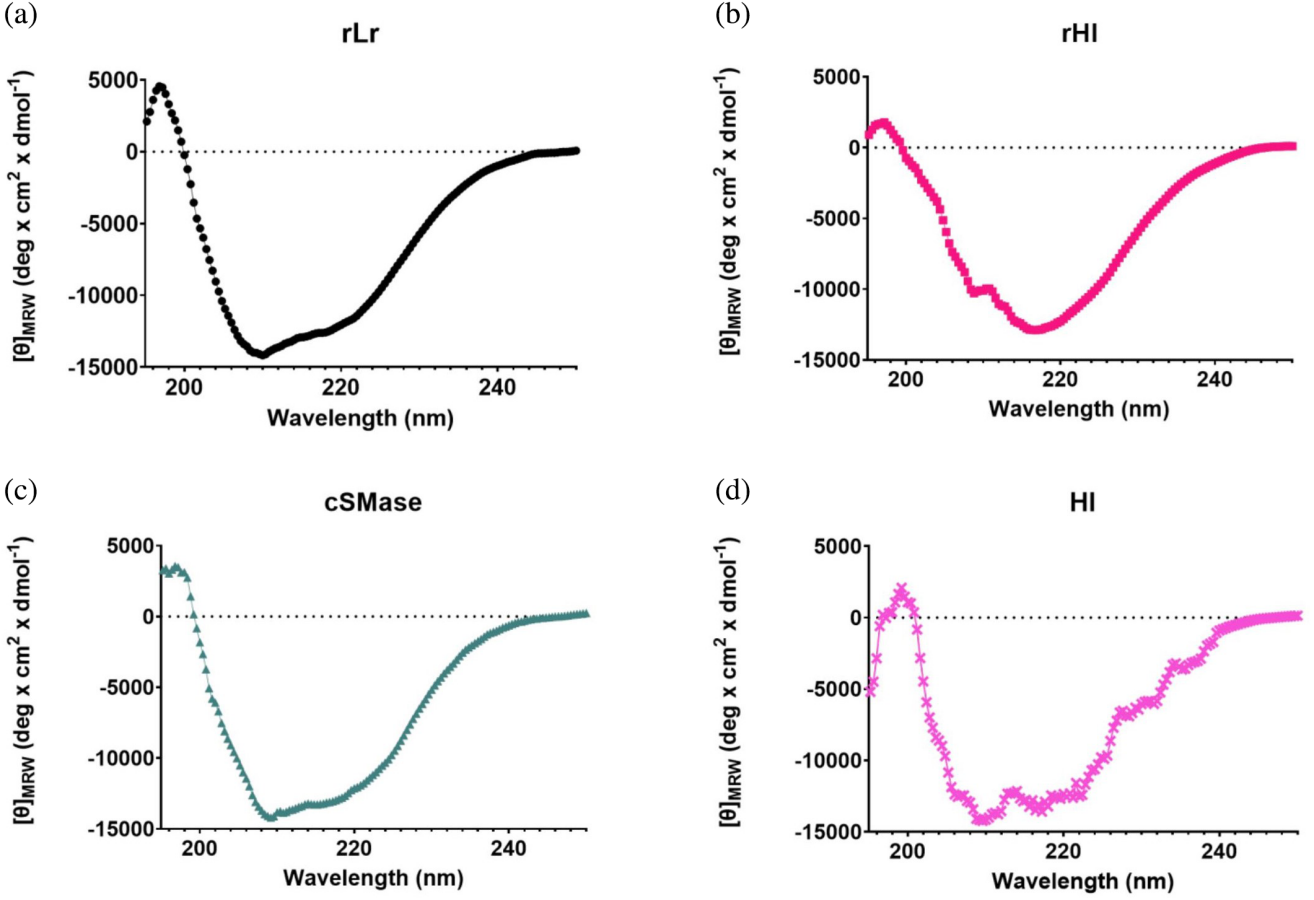
Far‐UV circular dichroism spectra of the recombinant proteins rLr (a, black dots), rHl (b, magenta squares), cSMase (c, green triangles), and Hl purified from *H. lepturus* venom (d, pink crosses). The ellipticity signal was converted to molar ellipticity per residue ([*θ*]_MRW_), considering the average amino acid weight to allow for comparison across spectra.

**FIGURE 3:**
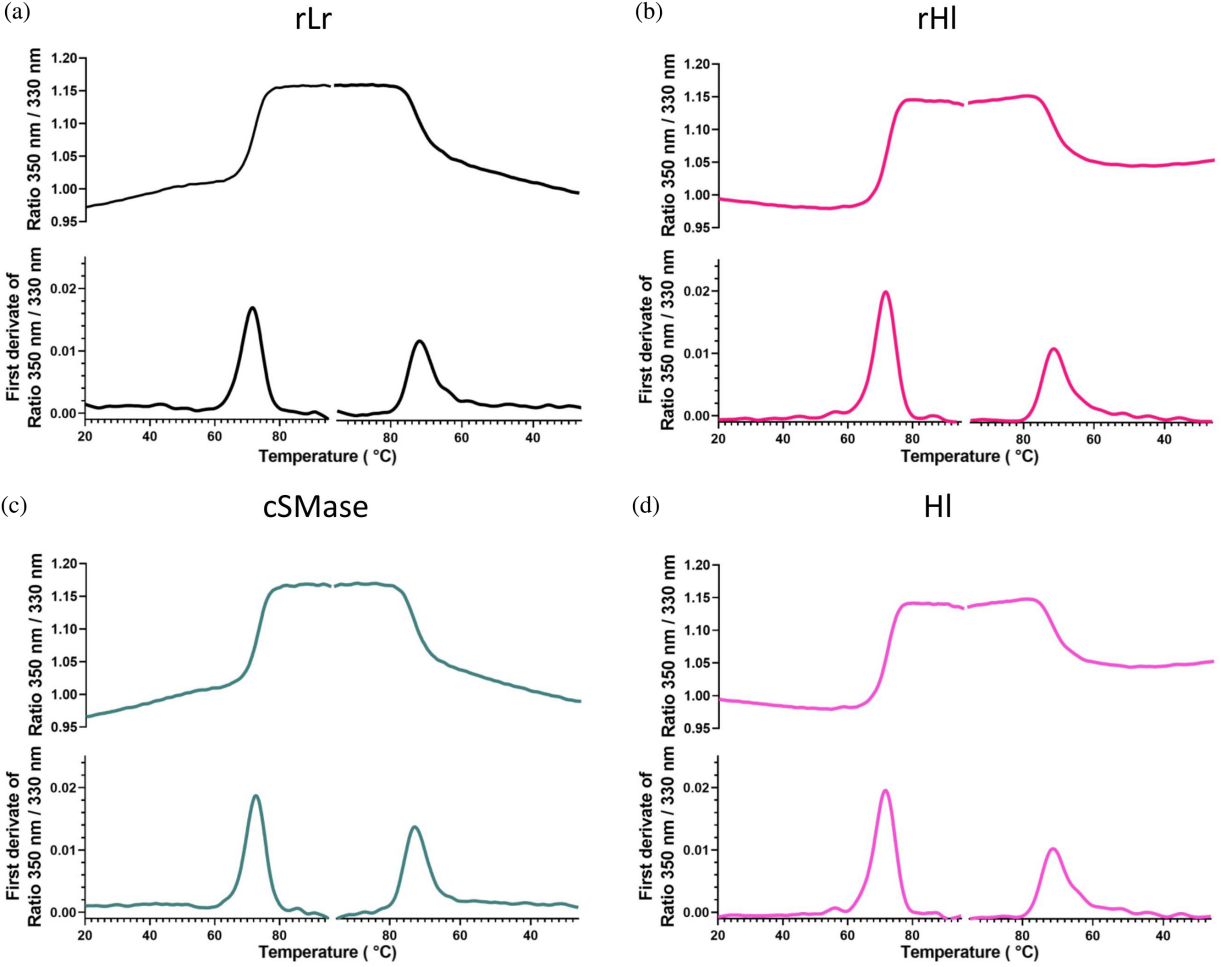
Thermal denaturation curves in NanoTemper for rLr (a, black), rHl (b, magenta), cSMase (c, green), and Hl (d, pink). The curves represent the average of three independent experiments. The samples were heated from 20°C to 95°C at a rate of 0.5°C/min and then cooled down to 20°C at the same rate. The first derivative of the denaturation/renaturation curves was calculated to determine the melting temperature.

For the design of a consensus sphingomyelinase (cSMase), a blastp search was performed on the NCBI database using the most representative queries from scorpions and recluse spiders. This search resulted in 136 unique sequences based on a non‐duplication and coverage criteria (>70%) (File S1). These sequences were aligned in Jalview 2, and a cSMase was designed based on the most commonly present amino acid or physicochemical property at each position (acid, basic, polar, or hydrophobic amino acid side chains). The signal peptides were manually trimmed from the final sequence to obtain the mature protein. Although the designed cSMase was not identical to any of the natural sequences, it shared 47.60% and 86.08% similarity with the most representative SMases from *H. lepturus* (A0A1L4BJ98) and *L. rufescens* (C0JB02.1), respectively (Figure 1b), which are the most prevalent Gadim scorpions and recluse spiders in the Middle East and North Africa region. Despite the differences in primary sequence, AlphaFold2 predicated that the three‐dimensional structures of the two natural SMases and the designed cSMase would be similar, featuring a β‐sheet barrel surrounded by α‐helices (Figure 1c). To further evaluate the consensus design and assess similarities across the 136 SMase sequences that were included in the design of the cSMase, a sequence identity matrix and a phylogenetic tree were made (Figure S1). The majority of sequences included in the design originated from *Loxosceles* spiders, while the remaining sequences were from other venomous species, including *Hemiscorpius* scorpions. Within the *Loxosceles* genus, the sequence variability was diverse with a maximum identity of 99.7% between isoforms from *L. intermedia* and a minimum identity of 39% between *L. similis* and *L. arizonica* (ANY30985 and Q7Z1Y7). For the *Hemiscorpius* genus, the SMases were more similar with the highest identity of 60.4% (API81381.1 and A0A1L4BJ98) and the lowest identity of 47.5% (API81379 and A0A1L4BJ98), both between *H. lepturus* species. Overall, the designed cSMase had the highest sequence identity to SMases from *L. hirsuta* (C0JAT6.1) recluse spiders with a score of 91%, and least identity to those from *H. lepturus* (API81379) with a score of 38%, underscoring that the cSMase most closely resembled SMases originating from recluse spiders compared to Gadim scorpions. The coding sequences of the *H. lepturus* (A0A1L4BJ98), *L. rufescens* (C0JB02.1), and the cSMase were optimized for bacterial codon‐usage and subcloned into an expression vector in phase with the 6xHis tag. The recombinant proteins were expressed in BL21(DE3) *E. coli* and purified to homogeneity with a single affinity chromatography step (Figure S2). The net expression yields were 3.0, 4.5, and 2.5 mg/L of culture for the recombinant SMase from *L. rufescens* (rLr), the recombinant SMase from *H. lepturus* (rHl), and the cSMase. *H. lepturus* venom was fractionated via HPLC, and fractions exhibiting electrophoretic mobility on SDS‐PAGE consistent with SMases were selected for further analysis. The recombinant proteins were subjected to analytical size exclusion chromatography, confirming the presence of a single peak per protein and indicating high purity, as observed by SDS‐PAGE (Figures S3 and S4). The elution volumes determined for rLr, rHl, and cSMase, allowed for the determination of the apparent molecular weight in a calibrated column resulting in 29.4 kDa, 31.4 kDa, and 31.5 kDa. For the native SMase, the recovery yield was approximately 150 μg per mg of crude *H. lepturus* venom (Hl). Circular dichroism spectra of the recombinant toxins and the SMase purified from *H. lepturus* venom showed that the pure proteins had a folded structure with a relative minimum ellipticity at 220 nm and an absolute minimum value around 208 nm, which is a hallmark of α‐helix‐rich structures (Figure 2). These spectra closely resembled those of native SMases purified from the crude venom of *Loxosceles* sp. (de Andrade et al., 2005). Thermal stability of the recombinant proteins was tested using a differential scanning fluorometer (NanoDSF), and the *T*_m_ values were determined by fitting the experimental data to a polynomial function. The slope maxima indicated by the peak of its first derivative resulted in 72.3°C, 71.63°C, and 72.6°C for rLr, rHl, and cSMase, respectively. All the proteins were able to refold, at least partially, upon cooling with *T*_m_ values equivalent to the first ones measured for all the proteins. The *T*_m_ for Hl was determined to be 72.6°C, and the protein was also able to refold upon cooling. The differences between the Hl and the recombinant proteins might be due to minor contaminations from other components of the venom in the fraction or posttranslation modifications that are absent in the recombinant proteins, which could influence in the thermal stability (Figure 3).

**FIGURE 4:**
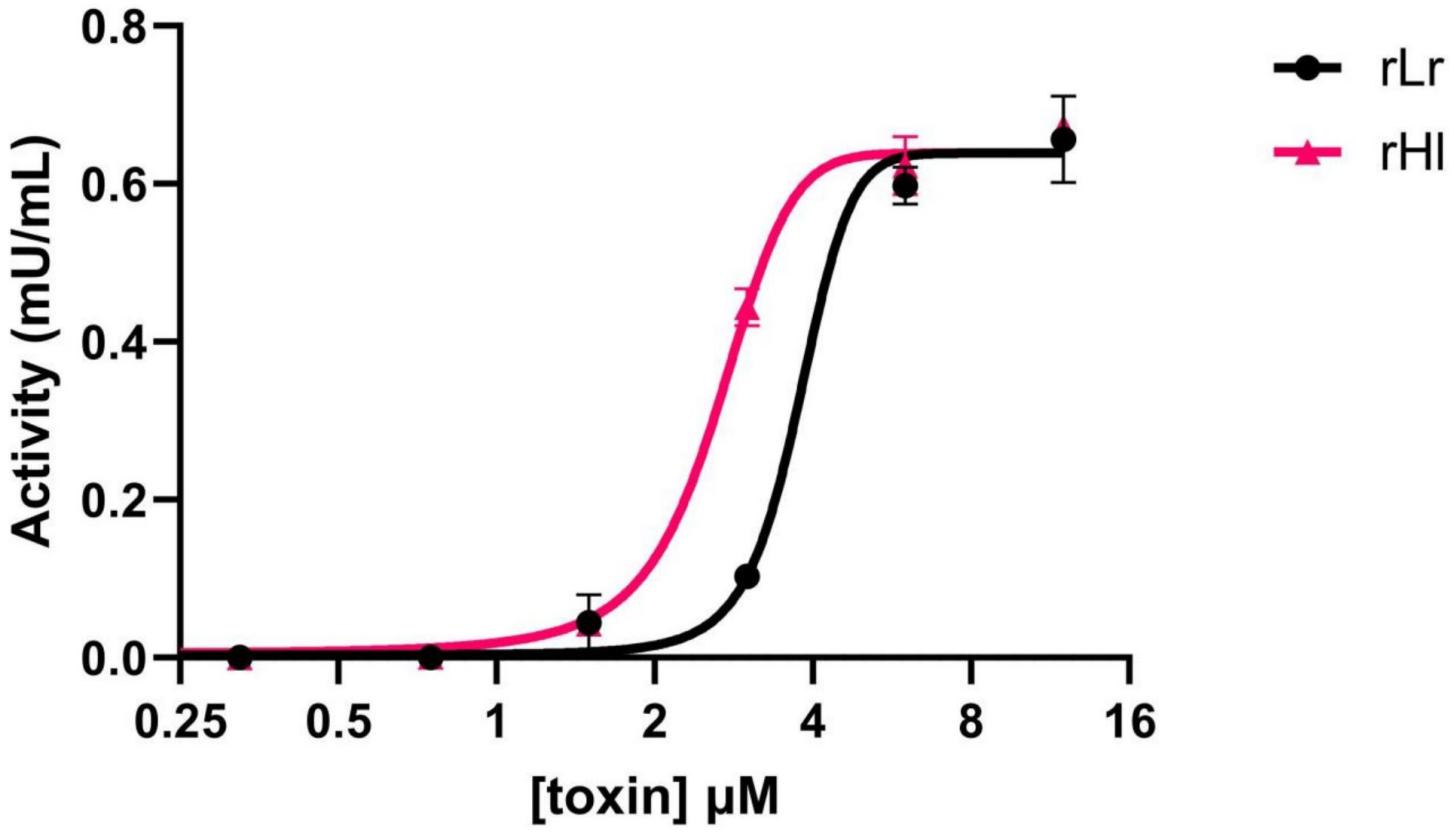
Sphingomyelinase activity of the natural recombinantly expressed toxins from *L. reclusa* (rLr, black circles) and *H. lepturus* (rHl, pink triangles). The points represent the average of triplicates, and the bars indicate the standard deviation of the measurement. Experimental data points were fitted to a nonlinear regression model.

Once the structural integrity of the samples was confirmed, the in vitro enzymatic activity of both rLr and rHl was evaluated and quantified (Figure 4). Under the tested conditions, both enzymes were active. Nonlinear regression analysis indicated that the EC_50_ values, which represent the toxin concentration required to achieve 50% of maximum activity, were 4.3 nM for rLr and 2.7 nM for rHl.

**FIGURE 5:**
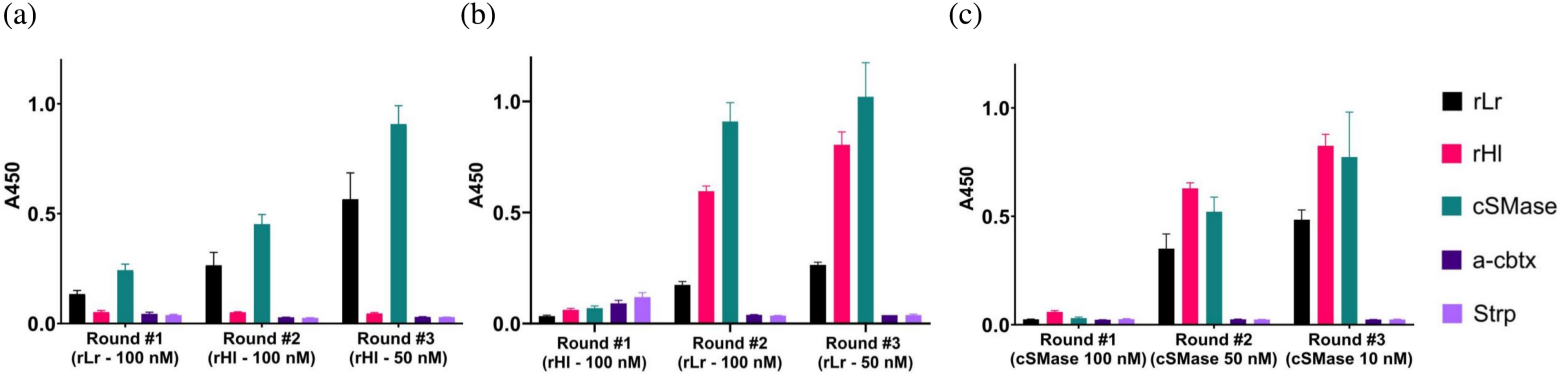
Polyclonal phage ELISA of the outputs from each phage display round (a–c). (a) Cross‐panning with recombinant toxins (rLr 100 nM, rHl 100 nM, rHl 50 nM). (b) Cross‐panning with recombinant toxins (rHl 100 nM, rLr 100 nM, rLr 50 nM). (c) Stringent panning with cSMase (100 nM, 50 nM, 10 nM). The bars represent an average of triplicates, and the error bars represent the standard deviation of the measurement. In each round, five different antigens were assayed: the three recombinant proteins rLr (black), rHl (pink), and the cSMase (green), and two negative controls: the native unrelated toxin α‐cobratoxin (α‐cbtx) (purple), purified from the venom of *Naja kaouthia*, and streptavidin (Strp) (lilac).

Upon characterization of the native and recombinant toxins, phage display technology was employed to select scFv antibody fragments from the IONTAS phage library κ with the aim of obtaining broadly‐neutralizing antibodies against both the scorpion and spider toxins. Two strategies were used to achieve this: cross‐panning with recombinant toxins (rLr and rHl) and stringent panning with the cSMase. To enrich the pool of phages cross‐binding to rLr and rHl, three rounds of cross‐panning were performed. In the first round, biotinylated rLr or rHl was used as antigen at concentrations of 100 nM. The antigen was alternated in the second round of panning but kept at the same concentration of 100 nM. The phages from the second rounds of selection were able to bind to the cSMase, but they did not show significant binding to the cross‐panned toxin, indicating a lack of cross‐binding of the antibody fragments displayed in the phage pools (Figure 5a,b). In the third round of selection, the concentration of the antigen used in the second round was reduced to 50 nM, resulting in a slight enrichment of phages binding to the target used in the first round as well as the cSMase. The selection campaign starting with rLr (Figure 5a) was enriched by 5.6× for rLr, 12.8× for the cSMase, and >1× for rHl in the second round, while for the third round, the relative enrichments were 2.2×, 2×, and >1.0× for rLr, the cSMase, and rHl, respectively. This confirmed that an enrichment of phages binding to the first toxin used in the discovery campaign had taken place. Following the same described tendency, the campaign involving cross‐panning starting with rHl (Figure 5b) showed an enrichment of 9.8× for rHl, 12.8× for the cSMase, and 5.6× for rLr, while for the third round, the enrichments with respect to the second round were 1.4×, 1.2×, and 1.5× for rHl, the cSMase, and rL respectively. Taken together, these results indicate that the campaign involving cross‐panning starting with rLr led to the selection of phage populations that can bind mainly to rLr. Similarly, the cross‐panning campaign starting with rHl primarily shows an enriched population of phages binding to rHl, although there might also be a second population of phages recognizing rLr or a population of cross‐reactive phages displaying antibodies that can bind both rLr and rHl. Nevertheless, output phages from both cross‐panning campaigns recognize the cSMase with a signal comparable to the recombinant toxins, indicating that the cSMase conserves structural features from both rLr and rHl.

The second strategy involved the use of the cSMase as the antigen for all three rounds of panning, with decreasing antigen concentrations in each round (100, 50, and 10 nM). This approach aimed to select for high‐affinity cross‐binders. The strategy resulted in an enrichment of phages displaying scFvs specifically binding to rLr, rHl, and the cSMase in the second and the third round (Figure 5c). Specifically, the enrichment folds were 17.5×, 20×, and 23× for rLr, rHl, and the cSMase, respectively, in the second round of selection, and 1.4×, 1.5×, and 1.5× in the third round of selection with respect to the second round. While these enrichment figures cannot be directly compared to those from the cross‐panning strategy due to the influence of antigen concentration on phage enrichment, the observed trend suggests that the use of the cSMase as antigen leads to a higher enrichment of binders for rLr and rHl, in contrast to the results observed using the cross‐panning strategies.

**FIGURE 6:**
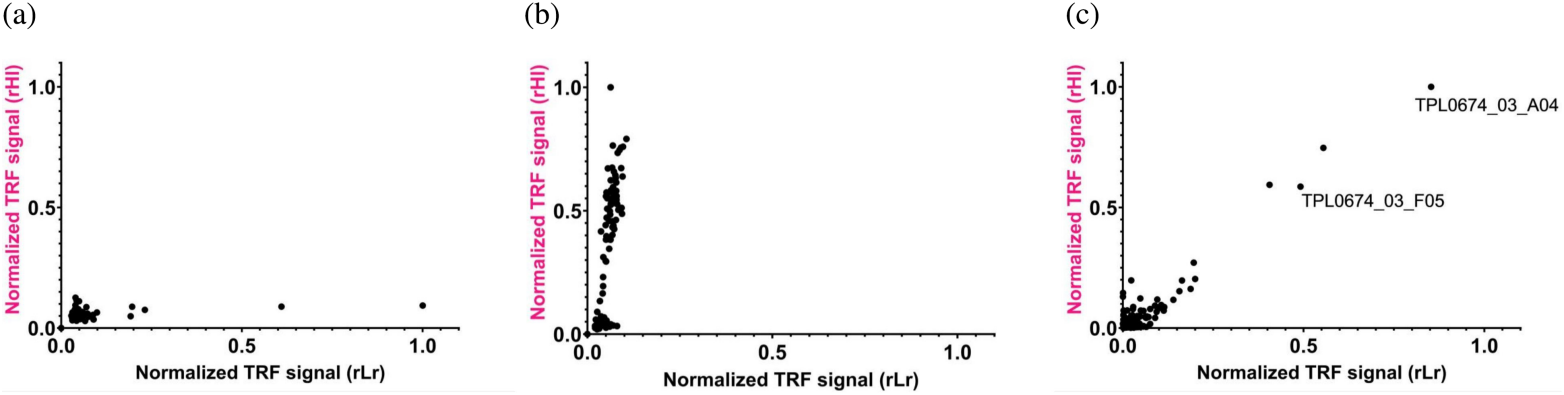
Monoclonal DELFIAs of the individual scFvs obtained by subcloning the last round of each discovery campaign described in Figure 4. Normalized TRF signal indicates binding to rLr (*x*‐axis, black) and rHl (*y* axis, pink). Each dot represents a single monoclonal scFv. (a) Cross‐panning with recombinant toxins (rLr 100 nM, rHl 100 nM, rHl 50 nM). (b) Cross‐panning with recombinant toxins (rHl 100 nM, rLr 100 nM, rLr 50 nM). Stringent panning with the cSMase (100 nM, 50 nM, 10 nM). Each black dot represents a single clone assayed for its binding to rLr and rHl. The two unique clones used for subsequent analysis are labeled in panel (c). scFvs, single‐chain variable fragments; TRF, time‐resolved fluorescence.

Upon completion of the selection campaign, the scFv‐encoding genes in the phages from the third panning round performed using either the cross‐panning strategies or using the stringent selection strategy with the cSMase as antigen were isolated, sub‐cloned into the pSANG10‐3F expression vector (Martin et al., 2006), and 276 clones were picked. The recombinant monoclonal scFvs were expressed in solution and tested for their ability to bind biotinylated rLr or rHl using a time‐resolved fluorescence (TRF) assay. The TRF signal, which is directly proportional to the binding strength of each scFv clone, was recorded and analyzed. The scatterplot in Figure 6 displays the normalized TRF signals for the ability of each scFv clone to bind to both recombinant toxins, rHl and rLr. The screened clones deriving from the campaign involving cross‐panning (Figure 6a,b) primarily only bound one of the antigens, coinciding with which antigen was employed in the first round of panning, in agreement with the results observed in the polyclonal phage ELISAs (Figure 5a,b). In contrast, the tendency is different for the clones deriving from the stringent selection campaign using the cSMase as antigen (Figure 6c), and it is worth noting that some scFvs showed equal binding intensities to both rHl and rLr. To identify the most optimal cross‐binding scFvs, a cut‐off value of 3 × 10^4^ TRF intensity over the negative control for both rHl and rLr and a TRF rLr/TRF rHl ratio between 0.8 and 1.2 was used (Table 1). As a result, two scFv binders were chosen for further characterization through DNA sequencing, resulting in two unique sequences (Figure S5). The scFvs, TPL0674_03_A04 and TPL0674_03_F05, were produced in mg scale and purified for further investigation of their binding affinity and neutralization capacity.

**TABLE 1:** Binders selected for sequencing.

Clone name	TRF rLr/TRF negative control	TRF rHl/TRF negative control	TRF rLr/TRF rHl
TPL0674_03_A04	7677	9004	0.85
TPL0674_03_F05	4424	5279	0.84

Abbreviation: TRF, time‐resolved fluorescence.

**FIGURE 7:**
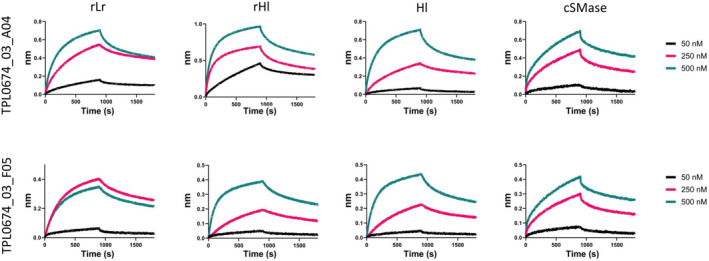
Binding curves measured by biolayer interferometry of TPL0674_03_A04 clone (top row) and TPL0674_03_F05 (bottom row) to rLr (first column), rHl (second column), Hl purified from crude venom (third column), and the cSMase (firth column). Each scFv was assayed at three different concentrations: 50 nM (black), 250 nM (pink), and 500 nM (green). cSMase, consensus sphingomyelinase; scFvs, single‐chain variable fragment.

**FIGURE 8:**
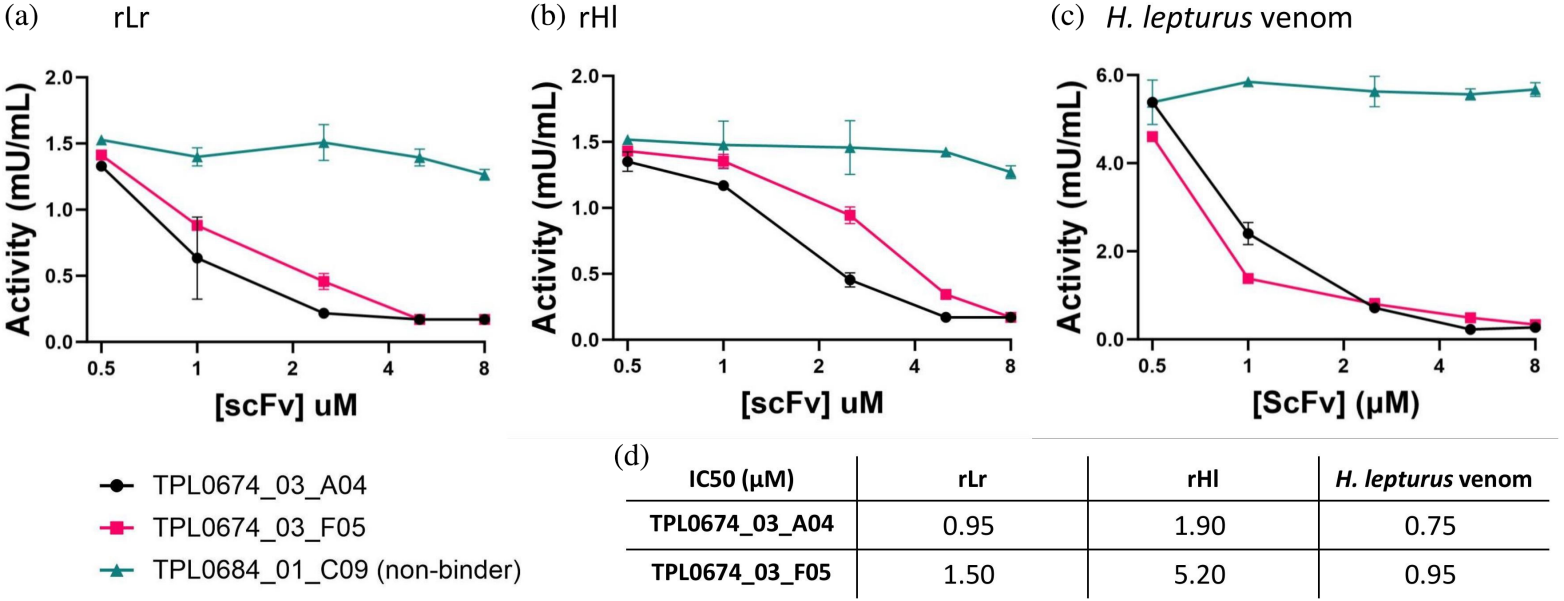
Neutralization of SMase activity of rLr (a), rHl (b), or *H. lepturus* whole venom (c) by TPL0674_03_A04 (black dots) and TPL0674_03_F05 (pink squares). An unspecific scFv (green triangles) was used as control. For the neutralization of rLr, the concentration used was 4.5 μM while for rHl, the concentration was 2.7 μM, corresponding with their calculated EC_50_ values (Figure 4). *H. lepturus* venom was assayed at 1.5 μg/mL. The table shows the calculated IC_50_ values, defined as the concentration of scFv necessary to inhibit 50% of the maximum activity registered in the assay.

To assess the binding affinity of cross‐reactive scFv antibodies against both recombinant SMases (rLr and rHl) and a biotinylated natural SMase purified from *H. lepturus*, biolayer interferometry was employed, demonstrating comparable binding affinities in the nanomolar range for both scFvs against the recombinant and natural toxins (Figure 7 and Table 2). To determine whether the cross‐reactive scFvs could neutralize the toxicity of the toxins, the enzymatic SMase activity of rLr, rHl, and *H. lepturus* whole venom was tested in the presence and absence of the scFvs at concentrations ranging from 0.5 to 8 μM. The toxin concentration chosen for the assay was the EC_50_ for rLr and rHl and 1.5 mg/mL for the *H. lepturus* venom. An irrelevant scFv (TPL0684_01_C09) was used as negative control. Both scFvs (TPL0674_03_A04 and TPL0674_03_F05) were found to successfully inhibit the SMase activity of the recombinant proteins as well as the *H. lepturus* venom, while the irrelevant scFv did not have any effect on the SMase activity (Figure 8). Finally, the IC_50_ values, defined as the concentration of scFv necessary to reduce the SMase activity with 50% of the total in the assay, were calculated (Figure 8d).

**TABLE 2:** Binding affinity determined by biolayer interferometry.

Clone	Toxin	*K*_D_ ± SD (nM)
TPL0674_03_A04	Hl	70.3 ± 0.9
	rLr	66.9 ± 0.8
	rHl	45.3 ± 0.6
	cSMase	31.5 ± 0.1
TPL0674_03_F05	Hl	60.7 ± 0.6
	rLr	80.4 ± 0.7
	rHl	51.6 ± 0.5
	cSMase	60.2 ± 0.4

Abbreviation: cSMase, consensus sphingomyelinase.

In summary, the results presented here demonstrate that the cSMase designed in this study could successfully be used as antigen in a phage display‐based discovery campaign to select cross‐reactive scFv antibodies (TPL0674_03_A04 and TPL0674_03_F05) that bind to recombinant and natural toxins with comparable affinity. These scFvs were found to specifically inhibit the SMase activity of *H. lepturus* venom, highlighting their potential as antibody leads for the development of broad‐spectrum toxin‐neutralizing agents. Based on these observations, we demonstrate that consensus proteins can be used as antigens during phage display selection campaigns for the selection of cross‐binding/neutralizing antibodies. While these results showcase the utility of this approach on SMases from scorpion and spider venoms, we speculate that this methodology might be applicable to other targets for the development of therapeutic antibodies.

## 3 DISCUSSION

In this study, we present the identification of several human monoclonal scFvs with broad neutralization capacity, obtained using phage display technology. The scFvs were selected against a recombinant toxin that was designed to mimic a consensus structure of the SMases produced by *Hemiscorpius* sp. and *Loxosceles* sp. The identified scFvs could bind to recombinantly produced version of native SMases from *H. lepturus* and *L. rufescens*, as well as a natural SMase purified from *H. lepturus* venom, with comparable affinities. Furthermore, the cross‐binding scFvs could neutralize the SMase activity of *H. lepturus* whole venom in vitro. We found that the selection strategy involving cross‐panning with alternating antigens (rLr and rHl) was not effective in generating broadly‐neutralizing scFvs for the target toxins we examined, as there was a lack of cross‐reactivity for the phage outputs. This suggests that the target toxins might not share enough epitope similarity to enable the obtainment of cross‐binders in a selection campaign involving cross‐panning, as rHl and rLr only share 43% sequence identity. However, we did find that the use of the cSMase, which represents the average of more than 100 native SMases, consistently enabled the enrichment of binding phages in each round of selection under stringent conditions, that is, increasing selection pressure through successive decreases in antigen concentration during consecutive panning rounds. The stringent selection conditions likely led to the selection of relatively high affinity scFvs with KDs in the nanomolar range (Table 2). However, when examining the IC_50_ values for the scFvs against rLr, rHl, and the cSMase, these fall within the μM range (Figure 8d). These relatively high IC_50_ values could potentially be explained by the fact that enzyme inhibition by an antibody is influenced by more than just binding affinity. Aspects like inhibition stoichiometry, the capacity of the antibody to disrupt the active site of the enzyme, and the reaction kinetics of the enzyme can all necessitate higher antibody concentrations for effective inhibition, as even a very small fraction of un‐neutralized enzyme may result in relatively high reaction rates depending on the assay conditions (e.g., if the rate of reaction close to the diffusion limit or far from this).

Beyond the results from this study, consensus toxins have previously been used to immunize rabbits and a horse and isolate serum containing polyclonal mixtures of antibodies displaying broad‐spectrum neutralization capabilities (de la Rosa et al., 2018; de la Rosa et al., 2019). However, it remains unclear if these broad neutralization capabilities derived from the induction of monoclonal bnAbs in the polyclonal mixture or if the broad neutralization capabilities derived from the polyclonality of the antiserum (Laustsen, 2021). Nevertheless, based on the data presented here, we can assert that consensus toxins can serve as powerful tools for obtaining monoclonal scFvs that can neutralize quite dissimilar antigens from distant sources, and we speculate that they can be used to generate monoclonal bnAbs both using in vitro and in vivo discovery approaches.

In relation to the potential therapeutic utility of the scFvs discovered here, it is important to understand that the clinical relevance of envenoming therapies against scorpion stings and spider bites is particularly significant in resource‐poor areas of the world (Jenkins et al., 2021). Diagnosing the specific spider or scorpion responsible for a bite can be challenging, underscoring the need for broadly effective envenoming therapies. Our study shows that the same scFvs can neutralize similar toxins from very distantly related species, which demonstrates their potential utility as a broadly‐neutralizing therapeutic agents for arachnid envenomings, if the results observed in vitro translate into the in vivo setting.

The use of consensus antigens, as presented here, can likely be extended to other areas, where the development of broadly‐neutralizing antibodies could be of therapeutic relevance. These areas include envenomings by other venomous animals, such as snakes or cone snails, infectious diseases, such as influenza or HIV, or hypermutating disease targets, such as those found in many cancers (Campbell et al., 2017; Wendel et al., 2020; Woodford and Ellington, 2007). Moreover, we envision that the use of consensus antigens may also find applications in combination with other established methods for the discovery and design of broadly‐neutralizing antibodies, including cross‐panning‐based phage display campaigns, structural approaches, and hybridoma and B‐cell screening. We thus hope that the results presented here may help expand the toolbox for the antibody discoverer.

## 4 MATERIALS AND METHODS

### 4.1 Consensus toxin design

Amino acid sequences for SMases from Hemiscorpiidae (scorpions) and Sicariidae (recluse spiders) families were collected from the National Center for Biotechnology Information (NCBI) website via blastp using as query the most representative members of the toxin family (A0A1L4BJ98 from *H. lepturus*, P0CE80 from *L. Intermedia*, P0CE79.1 from *L. reclusa*, and C0JB02.1 from *L. rufescens*) and default local alignment parameters. All hits were pooled and curated to eliminate duplicated entries. Multiple sequence alignments of the SMases were performed using the Jalview 2 program, with the signal peptides trimmed manually from all the sequences (Waterhouse et al., 2009). Sequences that overlapped <70% of the sequence length were eliminated from the pool. Consensus sequences were determined by conserving amino acid residues or physicochemical properties for each position.

### 4.2 Molecular modeling of cSMase structure

The three‐dimensional (3D) structures of cSMase and *L. rufescens* SMase were predicted by the state‐of‐the‐art AlphaFold2_mmseqs2 algorithm, which is part of the ColabFold set of Jupyter notebooks. The detailed methodology for this approach can be found in the respective literature (Jumper et al., 2021; Mirdita et al., 2022). Briefly, the amino acid sequence of cSMase was used as input for the AlphaFold2_mmseqs2 Jupyter notebook. This notebook is designed to predict protein structures using the AlphaFold2 algorithm, which combines machine learning with multiple sequence alignment (MSA) generation using the mmseqs2 method. The algorithm employs deep learning techniques to identify patterns in the input data and produce a high‐quality structural model. A predicted model of the *H. lepturus* SMase 3D structure was retrieved from the Alphafold Protein Structure Database (Varadi et al., 2022) and subjected to N‐terminal truncation of the first 45 residues using phenix.pdbtools (Liebschner et al., 2019).

### 4.3 Cloning

The coding sequences for the native SMases from *H. lepturus* (A0A1L4BJ98) and *L. rufescens* (C0JB02.1), as well as the designed cSMase were codon‐optimized for expression in *Escherichia coli* and purchased from Eurofins Genomics. All genetic constructs included the sequences *NcoI*‐6xHis and STOP‐*BamHI* in the 5′ and 3′ ends, respectively. The genetic constructs were subcloned into the pET 6xHis TEV cloning vector (Addgene #29653) using standard restriction and ligation cloning procedures. Briefly, both plasmid and genetic constructs were digested with *NcoI* and *BamHI* restriction enzymes (FastDigest, Thermofisher), inactivated (5 min at 65°C), and purified from agarose gel (GeneJET Gel Extraction Kit). Purified fragments were mixed in a 1:5 ratio (vector: insert) for ligation with T4 ligase, following the manufacturer recommendation (New England Biolabs). *E. coli* 10G Chemically Competent Cells (Lucigen) were transformed with the ligation mixtures and plated on LB plates containing 50 μg kanamycin (LB‐Kan) (Sigma‐Aldrich). Plasmids from four colonies with the expected electrophoretic pattern after analytical digestion with *NcoI*/*BamHI* (FastDigest, Thermofisher) were Sanger‐sequenced by Eurofins Genomics. The constructs were named pET‐6xHis‐rHl, pET‐6xHis‐rLr, and pET‐6xHis‐cSMase.

### 4.4 Expression and purification of recombinant toxins

Constructs of the recombinant SMases with confirmed sequence via Sanger sequencing were transformed by heat‐shock into *E. coli* BL21(DE3) competent cells for expression following the manufacturer's instructions (New England Biolabs). Colonies harboring pET‐rHl, pET‐rLr, and pET‐cSMase were selected based on their ability to grow on LB‐Kan plates (Sigma). Initial bacterial inoculum was grown overnight at 37°C in baffled Erlenmeyer flasks in LB‐Kan medium. Next, 500 mL of LB‐Kan was inoculated with 1/100 of the overnight culture. Expression was induced at OD_600_ of 1.0 with 1 mM isopropyl β‐D‐1‐thiogalactopyranoside for 12–14 h at 30°C with vigorous agitation. Then, cells were harvested by centrifugation at 5000 × *g* for 15 min at 4°C, and the cellular pellet was resuspended in 50 mM Tris pH 8.0, 20 mM imidazole. The cell suspension was subjected to seven pulses of sonication (20 kc, 1 min) in an ice bath. The water‐soluble fraction of this homogenate was recovered by centrifugation at 17,000 × *g* for 30 min at 4°C. Then, 1 mL of HisPur Ni‐NTA Resin (Thermo Fisher) equilibrated in resuspension buffer was added to the soluble fraction. The resin interacted with the water‐soluble fraction end‐over‐end rotation overnight at 4°C. The non‐retained fraction was collected, and the resin was washed with 50 mM Tris pH 8.0, 20 mM imidazole until absorbance at 280 nm was below 0.05. Finally, the recombinant toxins were eluted with five column volumes of 50 mM Tris pH 8.0, 250 mM imidazole. Milligram amounts of protein samples were purified to homogeneity according to their electrophoretic behavior analyzed by SDS‐PAGE. The recombinant toxins were dialyzed against PBS with 10,000 MWCO dialysis tubing (Snakeskin. Thermo Fisher), aliquoted, and flash‐frozen with N_2_(l) for long‐term storage at −20°C.

### 4.5 Reversed‐phase high‐performance liquid chromatography

The *H. lepturus* venom used in this study was provided by Seyed Mahdi Kazemi from Zagros Herpetological Institute and Mahboubeh Sadat Hosseinzadeh from the Department of Biology, Faculty of Sciences, University of Birjand, Birjand, Iran. To purify the native SMase from the venom, reversed‐phase high‐performance liquid chromatography (RP‐HPLC) was performed using an Agilent Infinity II (Santa Clara, CA, USA) system, as previously described (Calvete, 2011). Briefly, lyophilized venom (10 mg) was dissolved in 1 mL of water containing 0.1% trifluoroacetic acid (TFA; solution A), centrifuged at 14,000 × *g* for 10 min, and transferred to an HPLC vial. For each fractionation round, 100 μL of sample was injected into an RP‐HPLC C_18_ column (250 × 4.6 mm, 5 μm particle size) and eluted at 1 mL/min using a gradient towards acetonitrile containing 0.1% TFA (solution B) (0–15% B for 15 min, 15–45% B for 60 min, 45–70% B for 10 min, and 70% B for 9 min). Fractions with an absorbance response (280 nm) 10 times above the background were analyzed by SDS‐PAGE to detect the SMase based on its electrophoretic mobility. The fraction with the correct size was dialyzed against phosphate‐buffered saline (PBS: 137 mM NaCl, 3 mM KCl, 8 mM Na_2_HPO_4_⋅2H_2_O, 1.4 mM KH_2_PO_4_, pH 7.4) with 10,000 MWCO dialysis tubing (Snakeskin, Thermo Fisher) and flash‐frozen with N_2_(l) for long term storage at −20°C.

### 4.6 Size exclusion chromatography

Size exclusion chromatography was performed using a Superdex 75 Increase 10/300 GL column (Cytiva) equilibrated in phosphate‐buffered saline (PBS, pH 7.4). A total volume of 500 μL of the protein sample was injected into the column. The chromatographic separation was carried out with PBS as the running buffer, and the column was run over 1.5 column volumes to ensure adequate separation. Protein elution was monitored continuously by measuring the absorbance at 280 nm.

### 4.7 Near‐UV circular dichroism

Circular dichroism experiments were performed using a Jasco J‐810 circular dichroism spectropolarimeter, equipped with a Peltier‐controlled cuvette holder. Samples of recombinant proteins (rLr, rHl, and cSMase) at a concentration of 0.3 mg/mL in PBS were loaded into quartz cuvettes with a sample length of 1 mm prior to measurement.

### 4.8 Nanotemper

Protein denaturation analysis was conducted using a Nanotemper Prometheus NT.48 instrument, which employs microscale thermophoresis (MST) technology to monitor changes in protein fluorescence in response to temperature. Samples of proteins (1 μM in PBS) were heated from 20 to 95°C at a rate of 2°C/min, with changes in intrinsic protein fluorescence being measured using a NanoTemper capillary. Fluorescence data were collected at 1°C intervals, and the melting temperature (*T*_m_) was calculated using the Prometheus NT.48 software, which fits the data to a Boltzmann sigmoidal curve, and calculated based on four independent, averaged experiments.

### 4.9 Toxin biotinylation

The toxins were biotinylated using a 10:1 molar ratio of biotinylation reagent (Innolink Biotin 354S, Merck) to toxin as recommended by the manufacturer. Briefly, the biotinylation reagent was dissolved in dimethyl sulfoxide and added to the recombinant toxins in a small volume (<5% of the final volume). After 90 min of incubation at 25°C, biotinylated toxins were purified using Amicon® Ultra‐4 Centrifugal Filter Units with a 10 kDa membrane, with four washes using 4 mL PBS at 8°C. The protein concentration was determined by measuring absorbance at 280 nm with a NanoDrop and adjusted using the theoretical extinction coefficient calculated based on the protein sequence with ProtParam (Expasy) (Walker, 2005). The degree of biotinylation was analyzed by the *A*_280_/*A*_354_ absorbance ratio, resulting in a 2:1 to 3:1 biotin:toxin molar ratio for all the toxins tested.

### 4.10 Phage display selection

The IONTAS library, a human antibody phage display library of 4 × 10^10^ clones, was used for phage display selection. The library is constructed from B lymphocytes collected from 43 nonimmunized human donors, and contains antibodies in the form of scFvs (Laustsen et al., 2018). Before each round of selection, streptavidin‐specific phages were deselected using streptavidin‐coated Dynabeads (Invitrogen, M‐280). The biotinylated recombinant toxins were then incubated with the phage library in solution, which was blocked with 3% (w/v) skimmed milk in PBS (3MPBS). The biotinylated recombinant toxins were captured using streptavidin‐coated Dynabeads (Invitrogen, M‐280). After washing with PBS containing 0.1% (v/v) Tween 20 in a King Fisher Flex System, phages were eluted with trypsin and were added to cultures of *E. coli* TG1 cells with an OD_600_ of 0.5 and shaken at 150 rpm at 37°C for 1 h. Then, the cultures were plated and incubated overnight at 30°C on 2× TY medium plates (16 g/L tryptone, 10 g/L yeast extract, 5 g/L NaCl, 15 g/L agar) supplemented with 2% (w/v) glucose and 100 μg/mL ampicillin. The following day, the number of colony‐forming units was determined, and the plates were scraped using 2 mL of 2× TY medium (16 g/L tryptone, 10 g/L yeast extract, 5 g/L NaCl), supplemented with 2% (w/v) glucose and 50 μg/mL kanamycin and 25% (v/v) glycerol. The aliquoted phages were stored at −80°C.

Two panning strategies were compared: (1) stringent panning with cSMase, in which three rounds of panning were performed, while the antigen concentration was reduced in every round (100, 50, and 10 nM), and (2) cross‐panning with recombinant SMases from *H. lepturus* (rHl) and *L. rufescens* (rLr), in which the antigen was switched in the second and third round of panning, with the same antigen concentration of 100 nM in round 2, but with a lower antigen concentration of 50 nM in round 3 (i.e., rLr 100 nM, rHl 100 nM, rHl 50 nM, and rHl 100 nM, rLr 100 nM, rLr 50 nM). To recover phages from the selection outputs, 5 mL of 2× TY medium plates (16 g/L tryptone, 10 g/L yeast extract, 5 g/L NaCl) with 2% glucose and 100 μg/mL ampicillin were inoculated with the glycerol stock and grown at 37°C under shaking at 280 rpm until the OD_600_ reached 0.5. Then, the cultures were inoculated with 20× coverage M13KO7 helper phage (Jensen et al., 2018), which confers resistance to kanamycin, and incubated for 1 h at 37°C with shaking at 150 rpm. Then, the cultures were centrifuged at 3,500 × *g* for 10 min at 4°C, and the pellet was resuspended in 2× TY medium supplemented with 100 μg/mL ampicillin and 50 μg/mL kanamycin. The cultures were shaken overnight at 25°C at 280 rpm to produce phages. Rescued phages from every round were precipitated with 20% (w/v) PEG‐8000, 2.5M NaCl, followed by centrifugation and subsequent resuspension in PBS. Rescued phages were kept at 4°C for less than a week; 15% (v/v) glycerol was added to the phage aliquots for long term storage at −80°C.

### 4.11 Polyclonal phage ELISA

The selected phage outputs were evaluated for antigen binding using a polyclonal phage ELISA similarly to what has previously been described (Laustsen et al., 2018). In short, clear Maxisorp plates (Nunc) were coated with streptavidin (10 mg/mL) and biotinylated antigens (150 nM) were indirectly immobilized on the streptavidin‐coated wells. Negative control wells were coated with streptavidin or an irrelevant biotinylated toxin (α‐cobratoxin from *Naja kaouthia*), while positive control wells were coated with M13 helper phages in PBS. The plates were washed three times with PBS containing 0.1% (v/v) Tween 20 and then three times with PBS, before the addition of phage outputs from the different rounds in 3MPBS. After 1 h of incubation, plates were washed as before and 1:2,000 anti‐M13‐HRP antibody (Sino Biological #11973‐MM05T‐H) in 3MPBS was added to the wells. Following 1 h of incubation and a final washing step, the plates were incubated with 3,3′,5,5′‐tetramethylbenzidine (TMB) (Thermo Fisher #34021) for 15–20 min, followed by the addition of 0.5M H_2_SO_4_ to stop the reaction. Binding signals were detected by measuring the A_450_.

### 4.12 Subcloning

The scFv genes from the third selection round were amplified using PCR and subcloned using *NcoI* and *NotI* restriction endonuclease sites into the pSANG10‐3F vector for expression of soluble scFvs transformed into *E. coli* BL21(DE3) cells (New England Biolabs), as previously described (Ledsgaard et al., 2023; Rivera‐de‐Torre et al., 2022). The pSANG10‐3F vector contains a FLAG‐tag and a 6xHis tag in phase with the scFv. For each of the third selection rounds from the stringent consensus phage display campaign, 276 individual scFv clones were picked and incubated at 30°C and 800 rpm in 96‐well plates overnight in 1 mL of autoinduction medium. The next day, the scFv‐containing supernatants were tested for binding in an expression‐normalized capture DELFIA, as previously described (Laustsen et al., 2018).

### 4.13 scFv expression and purification

The TPL0674_03_F05 and TPL0674_03_A04 scFv clones were produced and purified as previously described (Ahmadi et al., 2020). Briefly, 2× TY medium, supplemented with 2% (w/v) glucose and 50 μg/mL kanamycin was inoculated with TPL0674_03_F05 and TPL0674_03_A04. The cultures were incubated overnight at 37°C and 250 rpm. The following day, 500 mL of autoinduction medium was inoculated with the overnight cultures and further incubated overnight at 30°C and 200 rpm. The cells were then harvested by centrifugation at 4300 × *g* for 10 min, and the supernatants were discarded. The cell pellet was resuspended in 50 mL of TES‐buffer (30 mM Tris–HCl pH 8.0, 1 mM EDTA, 20% sucrose (w/v)) containing 1.5 kU/mL of r‐lysozyme (~ 70,000 U/mg). After 20 min of incubation on ice, the cells were centrifuged at 4,300 × *g* for 10 min, and the supernatant was discarded. The cell pellet was resuspended in 50 mL of 5 mM MgSO_4_ supplemented with the same amount of r‐lysozyme as above and incubated on ice for 20 min. After centrifugation at 4300 × *g* for 10 min, the supernatant was pooled with the supernatant from the previous step and kept on ice. The pooled supernatants were then centrifuged at 30,000 × *g* for 30 min. The His‐tagged scFvs were purified using the same method as above for the recombinant toxins.

### 4.14 Expression‐normalized capture DELFIA

The binding of the scFvs to biotinylated rHl and rLr (30 nM) was assessed using a DELFIA‐based assay on black Maxisorp plates (Nunc), as previously described (Laustsen et al., 2018). Briefly, the scFvs were immobilized with anti‐FLAG M2 antibody (Sigma‐Aldrich #F3165) at 2.5 μg/mL. After immobilization of the scFvs, the biotinylated toxins (rHl and rLr) were added in 3MPBS. Binding was detected using streptavidin conjugated with europium (Perkin Elmer #1244‐360) and DELFIA Enhancement Solution (Perkin Elmer, 4001‐0010). To make the results from different experiments comparable, we included a positive control consisting of an antigen‐scFv pair with known positive binding (TPL 2552_02_B02 and α‐cobratoxin) (Ledsgaard et al., 2022). The TRF intensity values were then normalized to the TRF signal observed in this control.

### 4.15 Biolayer interferometry

Biolayer interferometry analysis was performed using a ForteBio Octet RED96 instrument with streptavidin tips. The tips were hydrated in kinetics buffer (Sartorius) for 30 min prior to use, then washed with PBS to remove any nonspecifically bound proteins. Biotinylated, recombinant SMase from *L. rusfescens* (rLr), recombinant SMase from *H. lepturus* (rHl), cSMase, or natural SMase purified from *H. lepturus* venom (Hl) were immobilized onto the tips in kinetics buffer at 50 nM for 10 min. Binding experiments were performed by dipping the streptavidin‐coated tips into a 96‐well microplate containing the purified scFvs at 50, 250, and 500 nM, followed by a dissociation step in kinetics buffer. Data analysis was performed using ForteBio Data Analysis software. The association and dissociation rate constants (*k*_a_ and *k*_d_) were calculated using a 1:1 binding model. The affinity constant (*K*_D_) was calculated as the ratio of *k*_d_ to *k*_a_. All data was analyzed in triplicate, and the average values were reported.

### 4.16 SMase enzymatic activity and neutralization assay

To evaluate the enzymatic activity of the recombinantly expressed toxins from *L. reclusa* (rLr) and *H. lepturus* (rHl), a ThermoFisher Colorimetric Sphingomyelinase Assay Kit (Catalog #MAK152‐1KT) was employed following the guidelines from the manufacturer. Specifically, a series of concentrations ranging from 0.15 to 12 μM of toxin was tested. The enzyme activity at these concentrations was then compared to a standard curve, generated using known sphingomyelinase (SMase) concentrations included in the kit. Using this data, nonlinear regression analysis was employed to calculate the EC_50_ value, which represents the toxin concentration needed to achieve 50% of the maximum enzymatic activity. The assay assesses the ability of the TPL0674_03_F05 and TPL0674_03_A04 scFvs clones to neutralize *H. lepturus* whole venom. The experiment was independently performed twice, with duplicates for each well. Briefly, the scFvs were diluted to concentrations ranging from 0.5 to 8 μM and then incubated with rLr, rHl, at the EC50 value or *H. lepturus* venom at a final concentration of 1.5 mg/mL. The mixture was combined with an assay reaction mix containing sphingomyelin and incubated for 2 h at 37°C. A SMase assay mixture containing alkaline phosphatase (ALP) and other enzymes was added to each well. The dephosphorylation of phosphorylcholine by ALP results in the production of a colored product that can be quantified by detecting the endpoint A_655_. The assay was run in parallel with a calibration curve, and negative controls included buffer alone and a nonbinding scFv (TPL0684_01_C09). The amount of sphingomyelinase activity present in the samples was determined from the standard curve.

## AUTHOR CONTRIBUTIONS

**Esperanza Rivera‐de‐Torre:** Conceptualization; investigation; methodology; validation; visualization; supervision; writing – review and editing; writing – original draft; project administration; formal analysis; resources. **Stefanos Lampadariou:** Investigation; validation; writing – review and editing. **Mark Møiniche:** Investigation; methodology; validation; visualization; writing – review and editing. **Markus F. Bohn:** Conceptualization; writing – original draft; writing – review and editing; project administration; formal analysis; investigation. **Seyed Mahdi Kazemi:** Resources; writing – review and editing. **Andreas H. Laustsen:** Writing – review and editing; conceptualization; funding acquisition; writing – original draft; investigation; project administration; resources; supervision.

## CONFLICT OF INTEREST STATEMENT

The authors declare no conflicts of interest.

## Supporting information


**Figure S1.** Comparison of SMases that were included in the design of the consensus SMase (cSMase). (a) Amino acid sequence identity matrix of selected sequences that represent the diversity of SMases included in the consensus design. The full identity matrix with all 136 sequences is included in File S2. (b) Phylogenetic tree with all 136 SMase sequences showing the evolutionary distance between SMases from different species. The tree was visualized with iTOL.

**Figure S2.** Chromatographic profile of *H. lepturus* venom, in which the absorbance at 280 nm is followed across the elution. The fraction containing the SMase toxin corresponding to the correct electrophoretic mobility (Figure S3d) is marked in red.

**Figure S3.** SDS‐PAGE of the purification process followed for the proteins used in the study. (a) This panel shows a representative SDS‐PAGE analysis of the purified recombinant toxins rLr, rHl, and the cSMase, obtained via affinity chromatography using HisPur Ni‐NTA Resin (Thermo Fisher). Different stages of the purification process are depicted, with a black square marking the fractions selected for further analysis. (b) This panel presents 15 μg of the recombinant proteins following 3‐month storage at −20°C. (c) SMases isolated from *H. lepturus* venom are shown, having been purified through HPLC fractionation (as outlined in Figure S2). (d) The purification steps for the selected scFvs are displayed, which were obtained by extracting the periplasmic fraction and subsequent purification via affinity chromatography using HisPur Ni‐NTA Resin (Thermo Fisher). Fractions enclosed by squares were pooled for ensuing experiments. The first well in all the SDS‐PAGE was loaded with 5 μL of Pre‐Stained Protein Ladder PageRuler (Thermo Fisher).

**Figure S4.** Size exclusion chromatography (SEC) of the recombinant toxins measured at 280 nm for (a) rLr, (b) rHl, and (c) the cSMase.

**Figure S5.** The figure presents the aligned amino acid sequences of the two scFvs, with TPL0674_03_A04 shown at the top and TPL0674_03_F05 at the bottom. Constant regions for both the heavy and light chains are highlighted in gray. Complementarity‐determining regions (CDRs) for the heavy and light chains are indicated in blue and yellow, respectively. Degree of sequence conservation is color‐coded: black for fully conserved amino acids, blue for conservation of physicochemical properties, and red for non‐conserved residues.

**File S1.** List of the 136 protein sequences used to design the cSMase sequence.

**File S2.** Percentage identity matrix displaying the 136 sequences used in the cSMase design as well as the cSMase.
