# Supplementary figures and images for: Discovery of broadly‐neutralizing antibodies against brown recluse spider and Gadim scorpion sphingomyelinases using consensus toxins as antigens
Source: Protein Sci. 2024 Feb 15;33(3):e4901. doi: 10.1002/pro.4901 (PMC10868436; doi:10.1002/pro.4901)

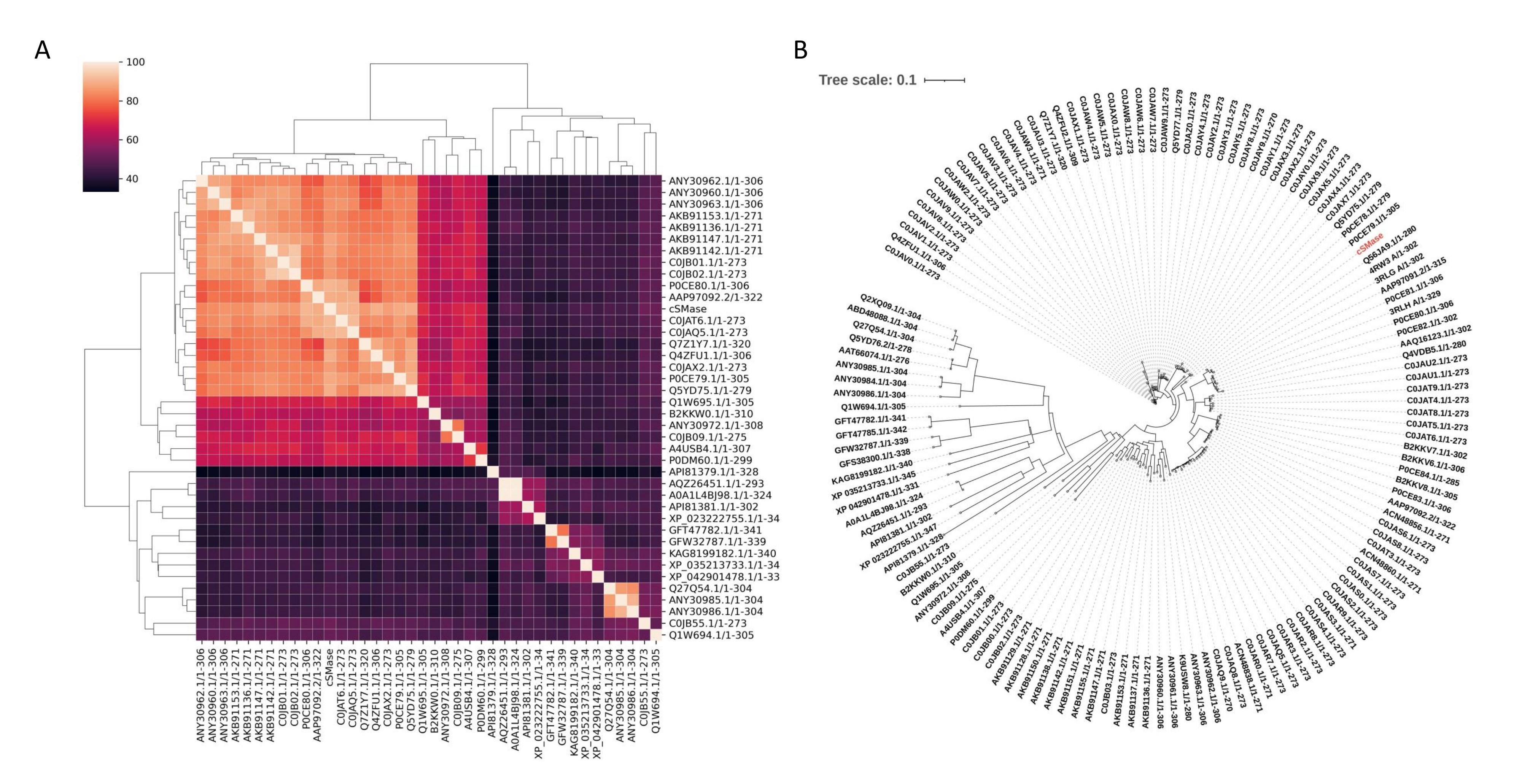

Supplement: Supplementary file 1 — Figure S1. Comparison of SMases that were included in the design of the consensus SMase (cSMase). (a) Amino acid sequence identity matrix of selected sequences that represent the diversity of SMases included in the consensus design. The full identity matrix with all 136 sequences is included in File S2. (b) Phylogenetic tree with all 136 SMase sequences showing the evolutionary distance between SMases from different species. The tree was visualized with iTOL. [file PRO-33-e4901-s002.jpg]

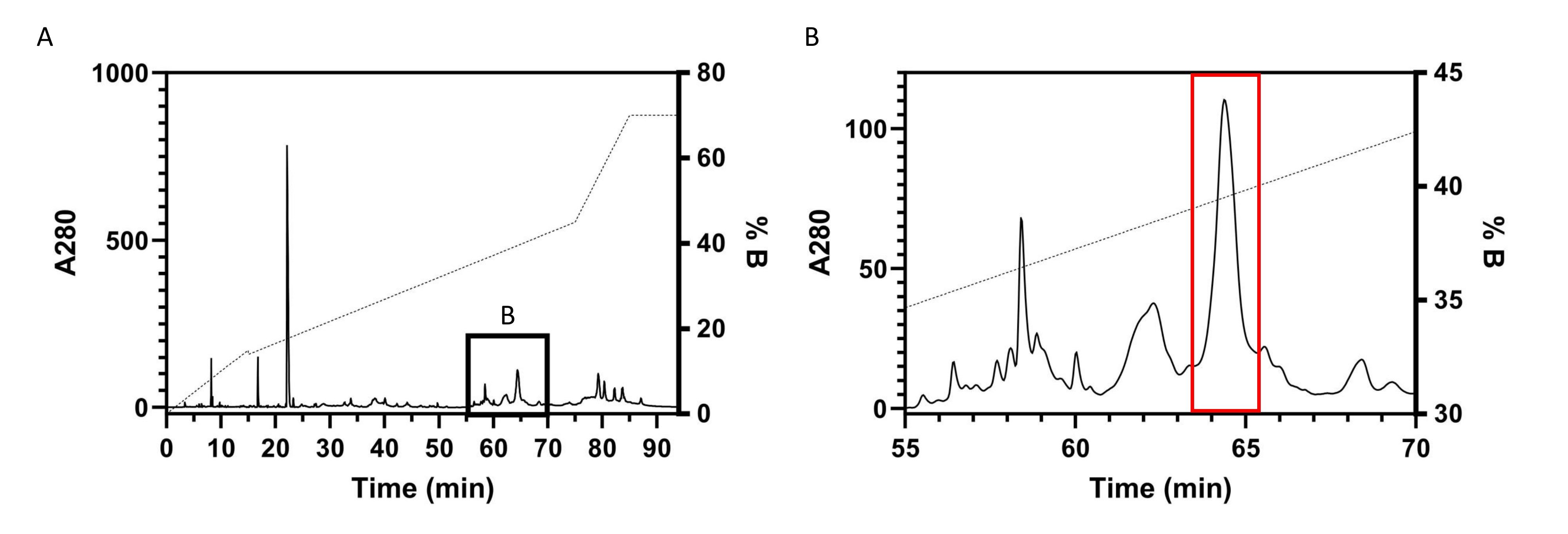

Supplement: Supplementary file 2 — Figure S2. Chromatographic profile of H. lepturus venom, in which the absorbance at 280 nm is followed across the elution. The fraction containing the SMase toxin corresponding to the correct electrophoretic mobility (Figure S3d) is marked in red. [file PRO-33-e4901-s005.jpg]

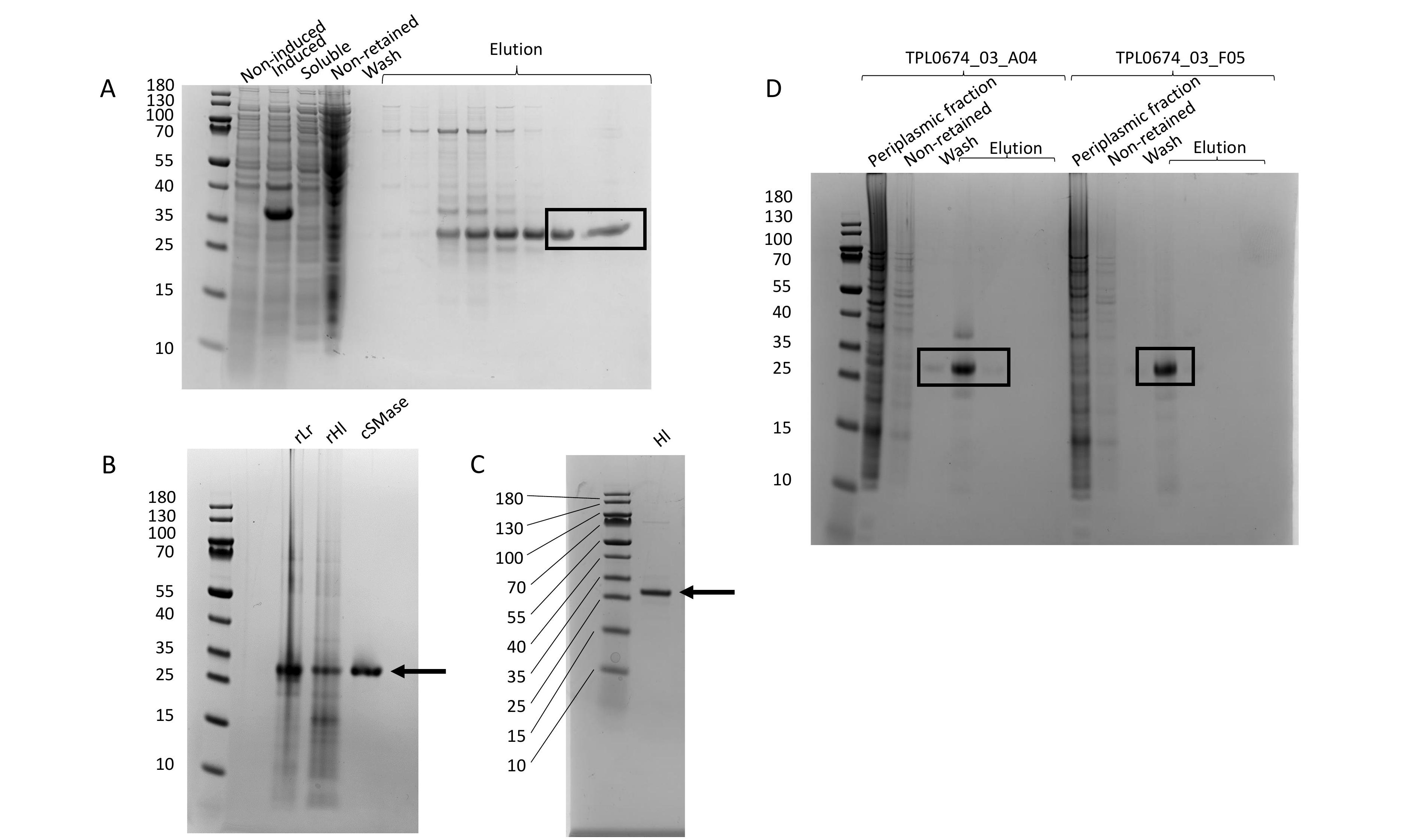

Supplement: Supplementary file 3 — Figure S3. SDS‐PAGE of the purification process followed for the proteins used in the study. (a) This panel shows a representative SDS‐PAGE analysis of the purified recombinant toxins rLr, rHl, and the cSMase, obtained via affinity chromatography using HisPur Ni‐NTA Resin (Thermo Fisher). Different stages of the purification process are depicted, with a black square marking the fractions selected for further analysis. (b) This panel presents 15 μg of the recombinant proteins following 3‐month storage at −20°C. (c) SMases isolated from H. lepturus venom are shown, having been purified through HPLC fractionation (as outlined in Figure S2). (d) The purification steps for the selected scFvs are displayed, which were obtained by extracting the periplasmic fraction and subsequent purification via affinity chromatography using HisPur Ni‐NTA Resin (Thermo Fisher). Fractions enclosed by squares were pooled for ensuing experiments. The first well in all the SDS‐PAGE was loaded with 5 μL of Pre‐Stained Protein Ladder PageRuler (Thermo Fisher). [file PRO-33-e4901-s004.jpg]

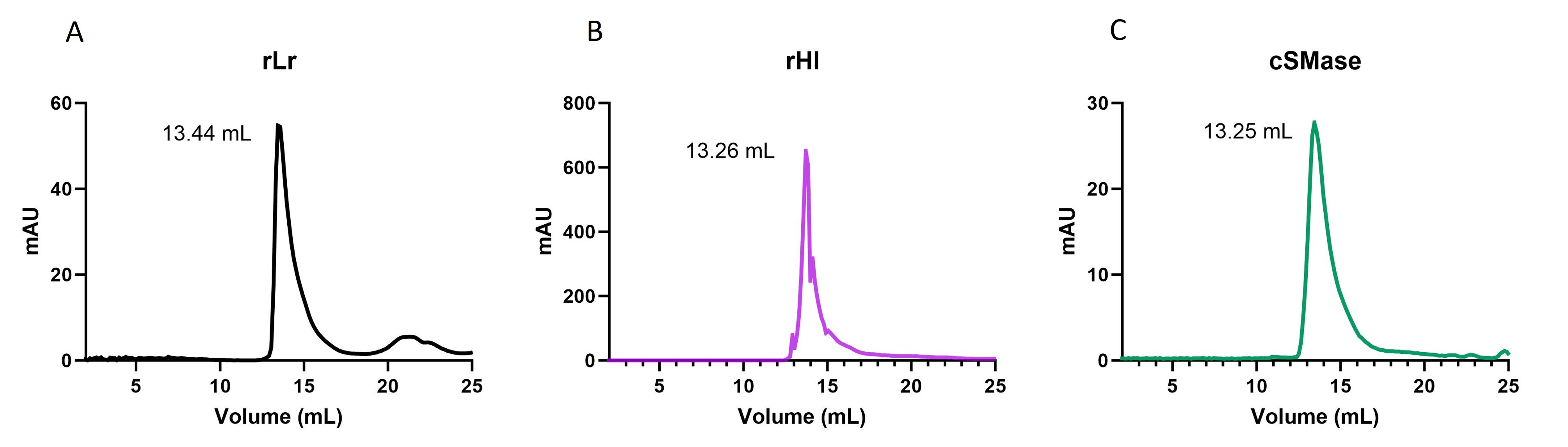

Supplement: Supplementary file 4 — Figure S4. Size exclusion chromatography (SEC) of the recombinant toxins measured at 280 nm for (a) rLr, (b) rHl, and (c) the cSMase. [file PRO-33-e4901-s003.jpg]

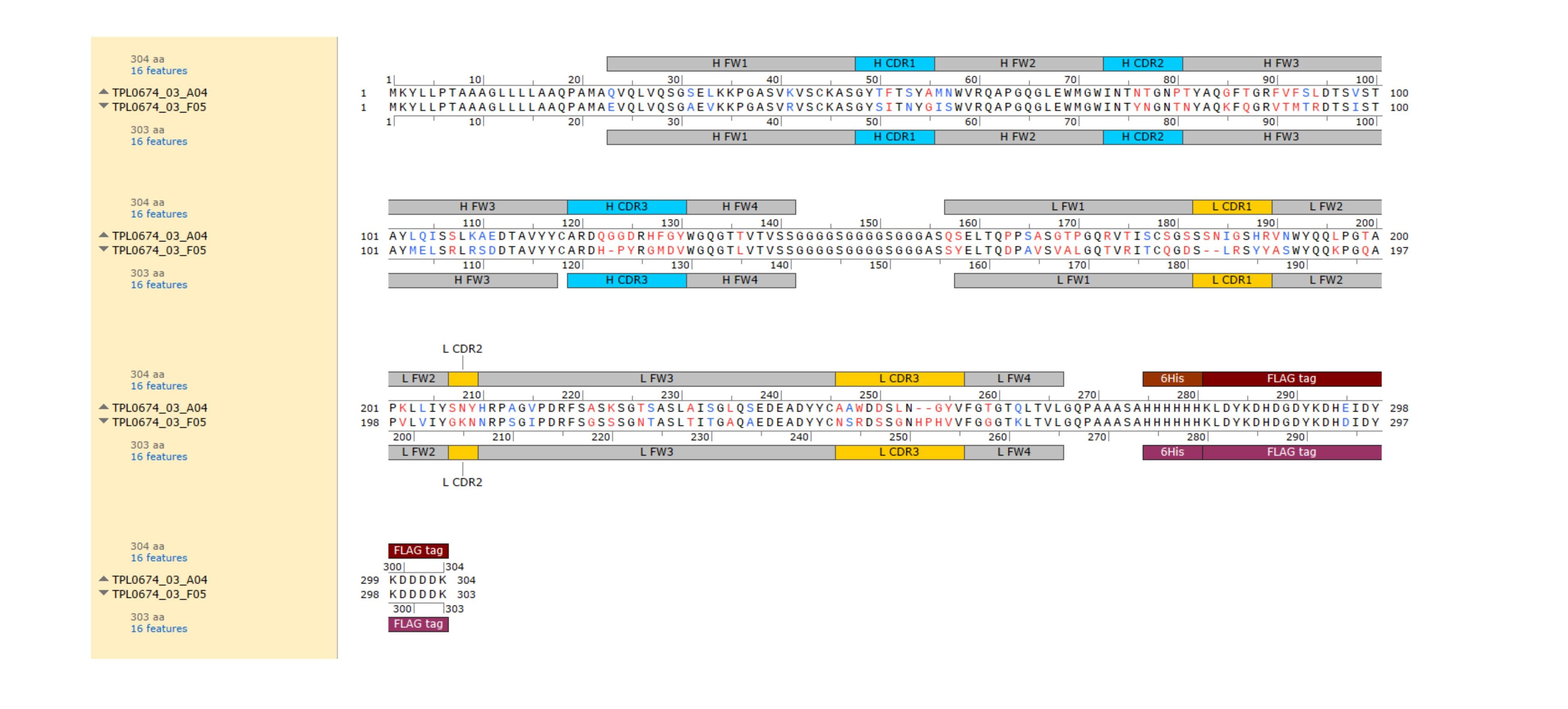

Supplement: Supplementary file 5 — Figure S5. The figure presents the aligned amino acid sequences of the two scFvs, with TPL0674_03_A04 shown at the top and TPL0674_03_F05 at the bottom. Constant regions for both the heavy and light chains are highlighted in gray. Complementarity‐determining regions (CDRs) for the heavy and light chains are indicated in blue and yellow, respectively. Degree of sequence conservation is color‐coded: black for fully conserved amino acids, blue for conservation of physicochemical properties, and red for non‐conserved residues. [file PRO-33-e4901-s006.jpg]
